# Impact of the Schottky Barrier and Contact‐Induced Strain Variations inside the Channel on the Electrical Behavior of Monolayer MoS_2_ Transistors

**DOI:** 10.1002/smsc.202500244

**Published:** 2025-09-28

**Authors:** Salvatore Ethan Panasci, Emanuela Schilirò, Giuseppe Greco, Patrick Fiorenza, Marilena Vivona, Salvatore Di Franco, Fabrizio Roccaforte, Fiorenza Esposito, Matteo Bosi, Giovanni Attolini, Igor Píš, Federica Bondino, Maddalena Pedio, Antonino Madonia, Marco Cannas, Simonpietro Agnello, Luca Seravalli, Filippo Giannazzo

**Affiliations:** ^1^ Consiglio Nazionale delle Ricerche Istituto per la Microelettronica e Microsistemi (CNR‐IMM) Strada VIII, 5, Zona Industriale I‐95121 Catania Italy; ^2^ Consiglio Nazionale delle Ricerche Istituto dei Materiali per l’Elettronica ed il Magnetismo (CNR‐IMEM) Parco Area delle Scienze 37/a 43124 Parma Italy; ^3^ Consiglio Nazionale delle Ricerche (CNR) Istituto Officina dei Materiali (IOM) Area Science Park, S.S. 14 Km. 163, 5, Basovizza I‐34149 Trieste Italy; ^4^ Department of Physics and Chemistry Emilio Segré University of Palermo Via Archirafi 36 90143 Palermo Italy

**Keywords:** doping, effective mass, liquid precursor chemical vapor deposition, MoS_2_, Schottky barrier, strains, transistors

## Abstract

Strain‐dependent electronic and optical properties are one of the most appealing features of 2D semiconductors, like monolayer (1L) MoS_2_. However, measuring and controlling the homogeneity of strain within the channel is crucial for next‐generation MoS_2_ field‐effect transistors (FETs). This article reports a multiscale investigation of backgated FETs fabricated using large‐area 1L MoS_2_ flakes grown by liquid‐precursor‐intermediated chemical vapor deposition on SiO_2_/Si substrates. The devices exhibit very attractive properties for ultra‐low power applications, such as an *I*_on_/*I*_off_ > 10^6^ and a normally off electrical behavior. The combination of temperature‐dependent analyses of the FET transfer characteristics and nanoscale resolution potential mapping by Kelvin probe force microscopy shows a fully depleted MoS_2_ channel at *V*_G_ = 0 and an effective Schottky barrier Φ_B,FB_ = 0.21 eV at flatband voltage *V*_FB_ = 17.9 V. An inhomogeneous tensile strain (*ε*) distribution along the channel length is revealed by micro‐Raman and photoluminescence (PL) mapping, with a reduced *ε* and blue‐shifted PL energy close to the Ni/Au source/drain contacts, suggesting a biaxial compression of 1L MoS_2_ induced by metal deposition. The implications of these observations on the effective mass m_eff_ variation along the channel and the current injection from source/drain contacts have been discussed in the perspective of future ultra‐scaled‐devices applications.

## Graphical abstract

This article reports a multiscale investigation of monolayer MoS_2_ field‐effect transistors, revealing a source/drain Schottky barrier of ≈0.21 eV and contact‐induced strain variations inside the channel. The impact of strain inhomogeneities on the electron effective mass (*m*_eff_) distribution and their implications on carrier mobility and contact resistance are discussed in the perspective of future ultra‐scaled transistor applications.

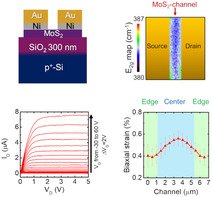

## 1 Introduction

Molybdenum disulfide (2H‐MoS_2_), the most common representative of layered transition metal dichalcogenides (TMDs),^[^ ^1^ ^]^ has been the object of widespread investigations during the last 15 years, owing to its peculiar properties like the thickness‐dependent bandgap ranging from 1.2 eV (indirect) for multilayers to 1.8–1.9 eV (direct) for monolayer (1L) MoS_2_.^[^ ^2^, ^3^ ^]^ These properties make MoS_2_ attractive especially as a channel material for ultra‐thin body field‐effect transistors (FETs), as well as for optoelectronic applications (photodetectors, solar cells, single photon sources), sensors, and new device concepts based on 2D materials or 2D/bulk semiconductors heterojunctions.^[^ ^4^, ^5^, ^6^, ^7^ ^]^ MoS_2_ FETs are especially strategic in the context of developing CMOS technologies beyond the current 3 nm technology node. In fact, scaling of Si transistors is currently reaching its physical limits, since further shrinking devices down to 1 nm technology node would imply reducing channel thickness <1 nm, with a consequent degradation of Si crystalline quality and channel mobility. Owing to its high carrier mobility (from ≈1 up to ≈200 cm^2^ V^−1^ s^−1^, depending on the synthesis method and passivation) in a ≈0.65 nm‐thick single crystalline film,^[^ ^8^ ^]^ MoS_2_ is considered as a potential replacement of Si to further extend Moore's law.^[^ ^9^ ^]^ As compared to competing 2D semiconductors, such as phosphorene and other Xenes, MoS_2_ has the advantage of being a naturally abundant material with good environmental stability. Indeed, highly scaled MoS_2_ channel transistor architectures have been reported in the last years to demonstrate the compatibility with semiconductor fabrication approaches, including arrays of MoS_2_ FinFETs,^[^ ^10^ ^]^ sidewall‐gated MoS_2_ channel transistors with <1 nm gate length,^[^ ^11^ ^]^ and multilevel integrated circuits and microprocessors made of MoS_2_ transistors.^[^ ^12^, ^13^, ^14^ ^]^

Owing to its broad range of applications, several approaches have been explored so far to produce MoS_2_ layers with high crystalline quality and controlled thickness uniformity on large area, including scalable top‐down methods, such as metal‐assisted exfoliation from bulk crystals,^[^ ^15^ ^]^ and bottom‐up methods, such as different chemical vapor deposition (CVD) approaches^[^ ^16^, ^17^, ^18^, ^19^, ^20^ ^]^ and metal‐organic‐chemical‐vapor deposition (MOCVD).^[^ ^21^, ^22^ ^]^ Among CVD approaches, liquid‐precursor‐intermediated chemical vapor deposition (LPI‐CVD) recently gained increasing interest as a cost‐effective method enabling improved scalability and better control on the thickness, morphology, crystallinity, and doping of deposited MoS_2_ for high‐performance FETs.^[^ ^20^, ^23^, ^24^, ^25^, ^26^, ^27^ ^]^

In spite of the many progresses achieved in the deposition techniques to improve MoS_2_ quality, the ultimate performances of MoS_2_ transistors are still limited by some material integration issues, such as the nonideal interfaces between MoS_2_ with back‐ and/or top‐gate insulators and source/drain contacts.^[^ ^28^, ^29^ ^]^ In particular, the reduction of contact resistance between metals and MoS_2_ represents one of the main challenges to achieve the theoretical performances of MoS_2_ transistors.^[^ ^30^ ^]^ Several investigations indicated disorder‐induced gap states (DIGS) at the interface between deposited metals and MoS_2_ as the origin of the commonly observed Fermi level pinning in this 3D/2D metal semiconductor system,^[^ ^31^, ^32^, ^33^ ^]^ and different strategies have been explored to circumvent this inherent issue. To eliminate metal deposition‐induced damage and interfacial reactions, the realization of van der Waals metal contacts onto MoS_2_ has been proposed by transferring the metal layer,^[^ ^32^ ^]^ by inserting a thermally decomposable buffer layer at the interface between the deposited metal and MoS_2_,^[^ ^34^ ^]^ or by evaporation of special gold‐capped indium (In) contacts.^[^ ^35^ ^]^ The insertion of a 2D layer (such as monolayer graphene or h‐BN tunnel barrier) between deposited contacts and MoS_2_ has also been demonstrated as an effective approach to achieve Fermi level depinning at the interface.^[^ ^36^, ^37^, ^38^ ^]^ Although van der Waals contacts to MoS_2_ look very promising to achieve very low contact resistance, their practical integration in scalable processes can be difficult. Hence, many efforts have been dedicated in the last years to optimize the deposition conditions of common CMOS‐compatible metals onto MoS_2_ (and other 2D materials) to minimize interface damage.^[^ ^39^, ^40^ ^]^ As an example, evaporation of common metals (like Ni) in ultra‐high‐vacuum conditions provides low‐resistance Ohmic contacts for 1L MoS_2_ transistors.^[^ ^38^ ^]^ Furthermore, recent studies focused on the impact of deposited metal contacts on the uniformity of strain distribution inside the channel of monolayer MoS_2_ FETs, and on the overall device electrical behavior.^[^ ^41^ ^]^ Such effects are expected to be critical for next‐generation ultra‐scaled FETs with shrinked channel length.

In this work, we report a multiscale investigation of backgated FETs fabricated on large‐area 1L MoS_2_ structures grown on SiO_2_/Si substrates by LPI‐CVD. Macroscopic electrical characterization of the device showed excellent saturation of the output characteristics (*I*_D_–*V*_D_), while a normally off electrical behavior (with a positive threshold voltage *V*_th_ = 36.5 V) and high *I*_on_/*I*_off_ > 10^6^ current ratio were observed from transfer (*I*_D_–*V*_G_) characteristics. In the subthreshold regime, the FET behavior was dominated by the gate bias‐dependent Schottky barrier between Ni/Au source/drain contacts and MoS_2_ channel (Φ_B_ = 0.78 eV at *V*_G_ = 0 V and Φ_B,FB_ = 0.21 eV at flatband voltage *V*_FB_ = 17.9 V). Spatially resolved electrical mapping of MoS_2_ FET by Kelvin probe force microscopy (KPFM) showed a nearly uniform surface potential distribution within the MoS_2_ channel, with a contact potential difference of ≈0.4 eV with respect to source/drain contacts. Micro‐Raman mapping of MoS_2_ channel revealed a spatially uniform *n*‐type doping in MoS_2_ channel, consistently with KPFM results. Interestingly, an inhomogeneous strain distribution, with higher tensile strain in the central region of the channel and a reduced tensile strain close to the contacts, was deduced from micro‐Raman maps and confirmed by microphotoluminescence (PL) spectroscopy.

## 2 Results and Discussion

**Figure 1:**
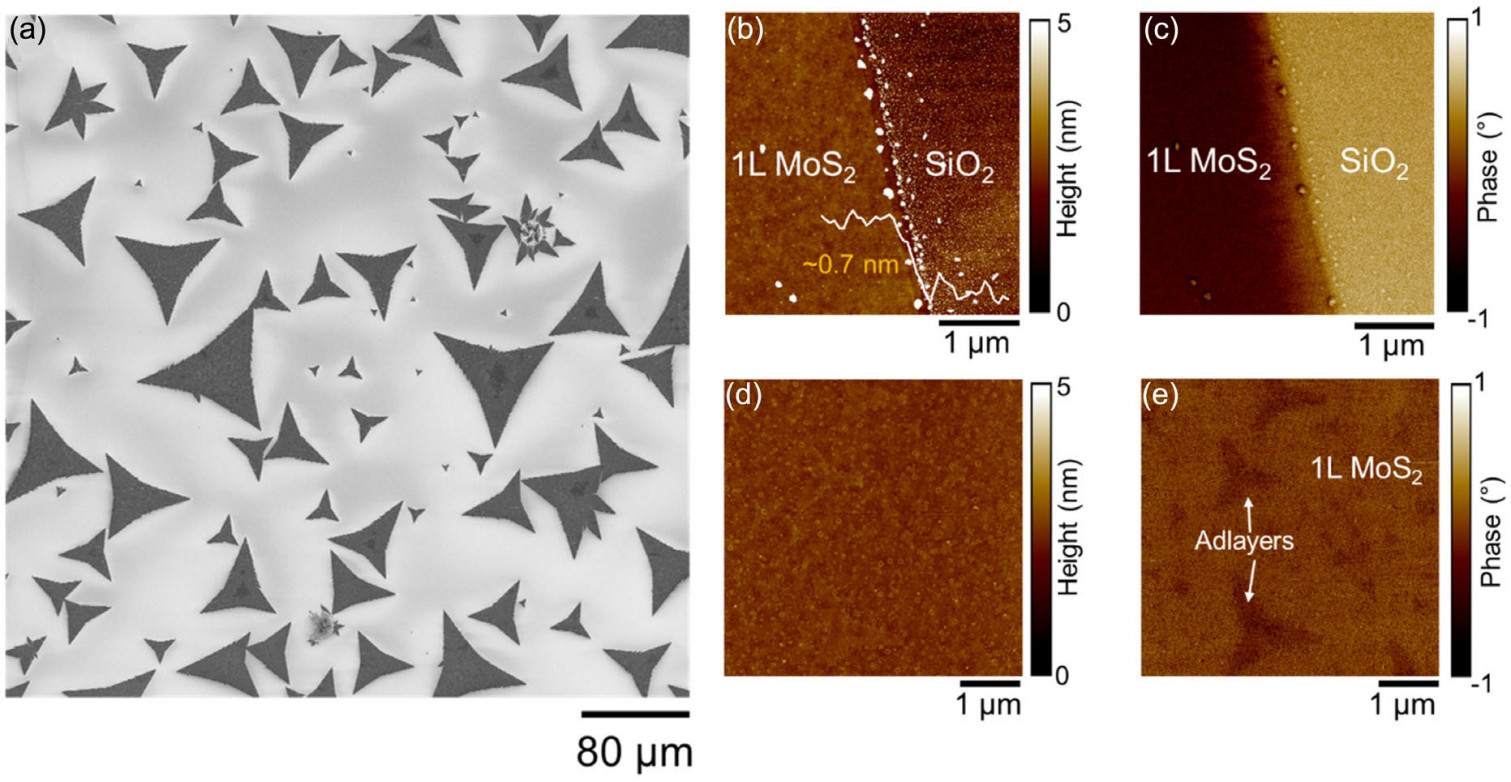
a) SEM image of the MoS_2_ flakes grown by LPI‐CVD on SiO_2_. b) Morphology and c) phase images collected at the edge of a MoS_2_ flake. The line profile in the inset of panel (b) displayed a step height of ≈0.7 nm corresponding to 1L MoS_2_ thickness. d) Morphology and e) phase images collected at the center of the triangular flake, displaying the presence of small MoS_2_ adlayers on the 1L MoS_2_ surface.

MoS_2_ samples have been grown on a SiO_2_(300 nm)/Si substrate by LPI‐CVD (with molybdenum liquid precursors) at 820 °C under S vapor flux, using as Mo precursor a spin‐coated solution of (NH_4_)Mo_7_O_24_ diluted in NaOH and mixed with iodixanol to increase viscosity in the solution.^[^ ^26^, ^42^ ^]^ This LPI‐CVD process results in the formation of a distribution of MoS_2_ flakes with nearly triangular shape and lateral size ranging between tens to hundreds of micrometers, as illustrated by the scanning electron microscopy (SEM) image in **Figure** **1**a. In this low‐resolution overview image, the uniform contrast indicates a homogeneous MoS_2_ thickness distribution inside the flakes, except for the presence of a central region with darker contrast (higher MoS_2_ thickness) observed for some of the triangular flakes. Figure 1b,c shows the tapping mode atomic force microscopy (AFM) morphology and phase images collected in the edge region of a large MoS_2_ flake, from which it is possible to distinguish the interface between the MoS_2_ and the bare SiO_2_ substrate. A step height of ≈0.7 nm, consistent with 1L MoS_2_ thickness, was deduced from the line profile across this edge (inset of Figure 1b). Furthermore, AFM morphology and phase maps in the central region of the flake (Figure 1d,e) revealed the nucleation of very small adlayers on the surface of the underlying 1L MoS_2_.

**Figure 2:**
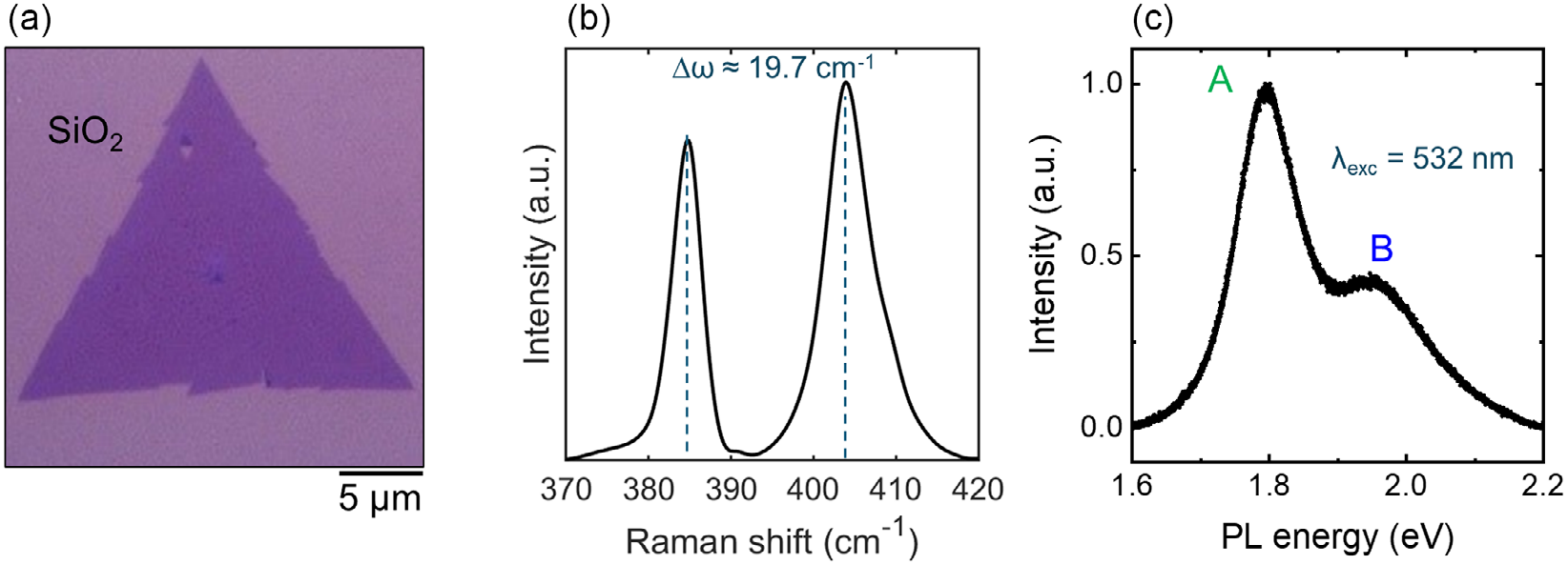
a) Optical image of a representative triangular MoS_2_ flake on SiO_2_. b) Raman spectrum acquired on the MoS_2_ flake, showing the first‐order vibrational modes E_2g_ and A_1g_. These two peaks displayed a frequency difference Δ*ω* ≈ 19.7 cm^−1^. c) Corresponding PL spectrum, acquired with an excitation wavelength of λ_exc_ = 532 nm, displaying a main exciton peak A at ≈1.77 eV and a lower intensity second peak B at ≈1.96 eV.

Micro‐Raman and micro‐photoluminescence spectroscopy were employed to investigate the vibrational and optical properties of the as‐grown MoS_2_. **Figure** **2**a shows the optical image of a flake, while a representative Raman spectrum is reported in Figure 2b, displaying the first‐order in‐plane (E_2g_) and out‐of‐plane (A_1g_) vibrational modes. Information about the MoS_2_ thickness can be extracted from the frequency difference (Δ*ω*) between these peaks, which was widely demonstrated to be dependent on the number of MoS_2_ layers.^[^ ^43^ ^]^ The obtained value of Δ*ω* ≈ 19.7 cm^−1^ is in perfect agreement with 1L MoS_2_ thickness, confirming previous AFM evaluation in Figure 1b. The E_2g_ and A_1g_ vibrational modes are very sharp (FWHM ranging between 3.4 and 4.5 cm^−1^) and exhibit a high intensity ratio (≈0.95), demonstrating a good crystalline quality of the CVD‐grown material.^[^ ^44^, ^45^ ^]^ The monolayer thickness and crystalline quality of MoS_2_ flakes are also confirmed by the representative PL spectrum in Figure 2c, displaying a main excitonic peak A at ≈1.77 eV and lower intensity peak B at 1.96 eV, associated with spin‐orbit splitting of the MoS_2_ valence band.^[^ ^2^, ^3^ ^]^

Besides representative Raman and PL spectra, full maps of the E_2g_, A_1g_ peak frequencies, their difference Δ*ω*, and the PL peak energy acquired on entire MoS_2_ flakes are also reported in Figure S1, Supporting Information.

**Figure 3:**
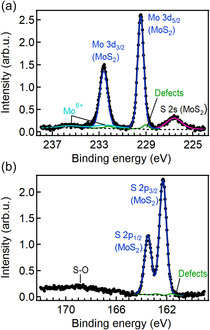
XPS spectra of a) Mo 3*d* and S 2*s* and b) S 2*p* core levels of 1L MoS_2_ flakes grown on a SiO_2_ substrate by LPI‐CVD.

X‐ray photoelectron spectroscopy (XPS) was carried out to analyze the chemical state and composition of the 1L MoS_2_/SiO_2_ surface. **Figure** **3** shows high‐resolution XPS spectra in the Mo 3*d* and S 2*p* core level regions. The main Mo 3*d* and S 2*p* peaks correspond to Mo^4+^ and S^2−^ in MoS_2_.^[^ ^46^ ^]^ The narrow peak widths indicate good crystalline quality, although not as high as that of 1L MoS_2_ exfoliated from a bulk MoS_2_ single‐crystal (Figure S2, Supporting Information). A slight asymmetry toward lower binding energies can be attributed to the presence of a low density of single‐atom structural defects in MoS_2_, such as sulphur vacancies.^[^ ^47^ ^]^ Additional weak Mo 3*d* peaks at higher binding energies are related to the Mo^6+^ oxidation state in molybdenum oxides. This may indicate partial oxidation of MoS_2_ upon exposure to air or the possible presence of residual sodium molybdate (Na_2_MoO_4_) and its complex with silicon, formed during thermally induced reactions between the Mo precursor, NaOH promoter, and the SiO_2_ substrate.^[^ ^48^ ^]^ Noteworthy, the presence of Na atoms was confirmed by both photoelectron and X‐ray absorption spectroscopy (Figure S3, Supporting Information). No other elements were detected on the sample surface, except for carbon. XPS also confirmed the absence of iodine, which could have remained as a residue from the decomposition of the iodixanol density gradient. The possible role of byproducts from LPI‐CVD reactions on the electrical behavior of MoS_2_ FETs will be discussed in the following.

**Figure 4:**
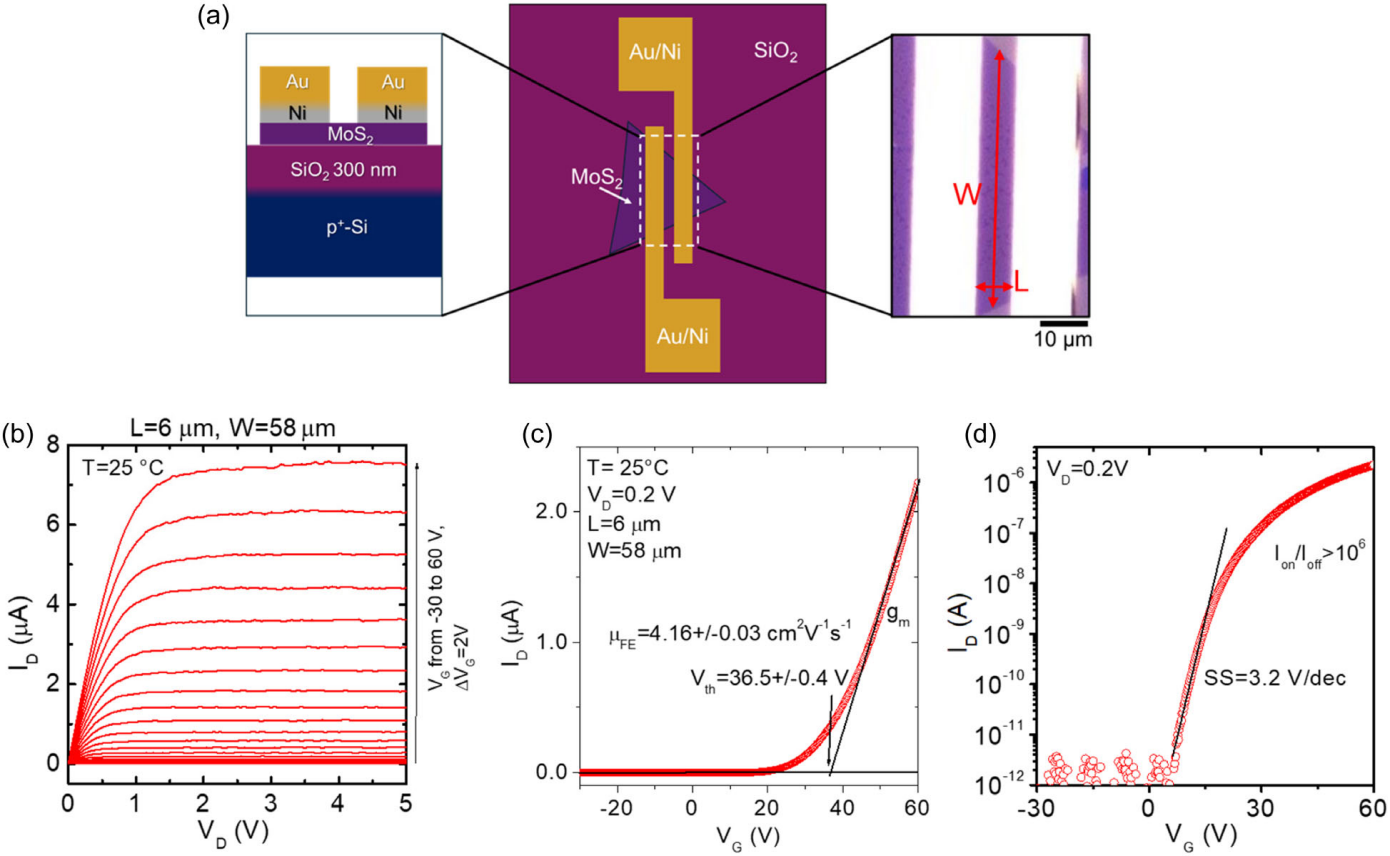
a) Cross‐sectional and top‐view schematic illustrations, and optical microscopy of 1L MoS_2_ transistor with a SiO_2_ (300 nm)/Si back‐gate and Ni/Au source and drain contacts (channel length *L* = 6 μm and width *W* = 58 μm). b) Output characteristics *I*_D_–*V*_D_ for *V*_G_ from −30 to 60 V (with steps Δ*V*_G_ = 2 V), showing Ohmic behavior at low *V*_D_ (<1 V), followed by saturation plateaus at higher *V*_D_. c) Linear scale and d) semilog‐scale transfer characteristics *I*_D_–*V*_G_ at *V*_D_ = 0.2 V acquired at room temperature (*T* = 25 °C). The field‐effect mobility *μ*_FE_ = 4.16 cm^2^ V^−1^ s^−1^ and threshold voltage *V*_th_ = 36.5 V have been evaluated from the linear scale *I*_D_–*V*_G_ curve, while an *I*_on_/*I*_off_ > 10^6^ and an SS = 3.2 V dec^−1^ have been extracted from the semilog *I*_D_–*V*_G_ characteristic.

After a preliminary assessment of the crystalline and optical quality of LPI‐CVD‐grown 1L MoS_2_, back‐gated FETs have been fabricated by thermal evaporation under high vacuum (10^−7^ mbar) of Ni (20 nm)/Au (80 nm) source/drain contacts. Top‐view and cross‐section schematics and a representative optical microscopy image of a device with channel length *L* = 6 μm and width *W* = 58 μm are shown in **Figure** **4**a. The transistor output characteristics, that is, the drain current (*I*_D_) versus drain bias (*V*_D_) for different gate voltage (*V*_G_) values from −30 to 60 V (with steps of Δ*V*_G_ = +2 V), acquired at room temperature (*T* = 25 °C), are reported in Figure 4b, showing very good Ohmic behavior at low *V*_D_ values (<1 V), followed by current saturation at higher *V*_D_. Furthermore, Figure 4c,d shows the room temperature transfer characteristics (*I*_D_–*V*_G_) at low drain voltage (*V*_D_ = 0.2 V), reported on linear and semilog‐scale, respectively. The monotonic increase of *I*_D_ as a function of *V*_G_ indicates an *n*‐type transistor behavior, that is, the formation of an electron accumulation channel above a threshold voltage (*V*_th_), as commonly reported for MoS_2_ FETs. In particular, a *V*_th_ = 36.5 ± 0.4 V was determined in Figure 4c as the intercept of the linear fit of the *I*_D_–*V*_G_ characteristic with the *V*_G_ axis. Noteworthy, the positive value of *V*_th_, corresponding to a “normally‐off” transistor at *V*_G_ = 0 V, represents a key aspect for low power consumption digital applications. A similar behavior, recently observed in back‐gated FETs based on MoS_2_ flakes grown on SiO_2_ surface using Au nanoparticles catalysts,^[^ ^49^ ^]^ was ascribed to electron transfer from MoS_2_ to Au nanoparticles driven by the work function difference between the two materials. In the present case of LPI‐CVD‐grown MoS_2_, a role in the observed normally off behavior can be ascribed to byproducts from reactions between the Mo precursor, NaOH promoter, and the SiO_2_ substrate, as discussed in the XPS analyses of Figure 3 and Figure S3, Supporting Information.

Furthermore, a field‐effect mobility *μ*_FE_ = 4.16 ± 0.03 cm^2^ V^−1^ s^−1^ has been estimated from the slope *g*_m_ = d*I*_D_/d*V*_G_ of the linear‐fit in Figure 4c according to the relation

(1) $$\mu =\frac{Lg_{m}}{WC_{\text{ox}}V_{D}}$$

where *C*_ox_ = *ε*_0_ *ε*_ox_/*t*_ox_ = 11.5 nF cm^−2^ is the capacitance per unit area of the SiO_2_ gate oxide (*t*_ox_ = 300 nm), *ε*_0_ is the vacuum permittivity, and *ε*_ox_ = 3.9 the oxide relative permittivity. Such mobility value is in line with typically reported values for not‐encapsulated 1L MoS_2_ obtained by exfoliation or by CVD growth.^[^ ^8^ ^]^ The semilog‐scale transfer characteristic in Figure 4d provides complementary information on the subthreshold behavior of the MoS_2_ FET, showing an off‐state drain current (*I*_off_) corresponding to the instrument detection limit (*pA*) for *V*_G_ < 5 V, a high on‐state versus off‐state current ratio *I*_on_/*I*_off_ > 10^6^, and a subthreshold swing SS = 3.2 V dec^−1^. The SS value, a measure of the FET switching efficiency, is expressed as $\text{SS}=\frac{\ln\left(10\right)k_{B}T}{q}\left(1+\frac{C_{s}+C_{\text{it}}}{C_{\text{ox}}}\right)$, being *k*_B_ the Boltzmann constant, *T* the temperature, *q* the electron charge, *C*_ox_ and *C*_s_ the capacitances of the gate oxide and 1L MoS_2_ semiconductor, and *C*_it_ = *qD*_it_ the capacitance contribution associated with the density of traps (*D*_it_) at MoS_2_/oxide interface. As compared to an ideal FET with ultra‐thin gate oxide and negligible *D*_it_, where the subthreshold swing value at *T* = 298 K approaches the Boltzmann limit SS = ln(10)*k*_B_ *T*/*q* ≈ 60 meV dec^−1^, the higher SS value of our 1L MoS_2_ FETs is accounted for by the thick (*t*_ox_ = 300 nm) back‐gate oxide.

**Figure 5:**
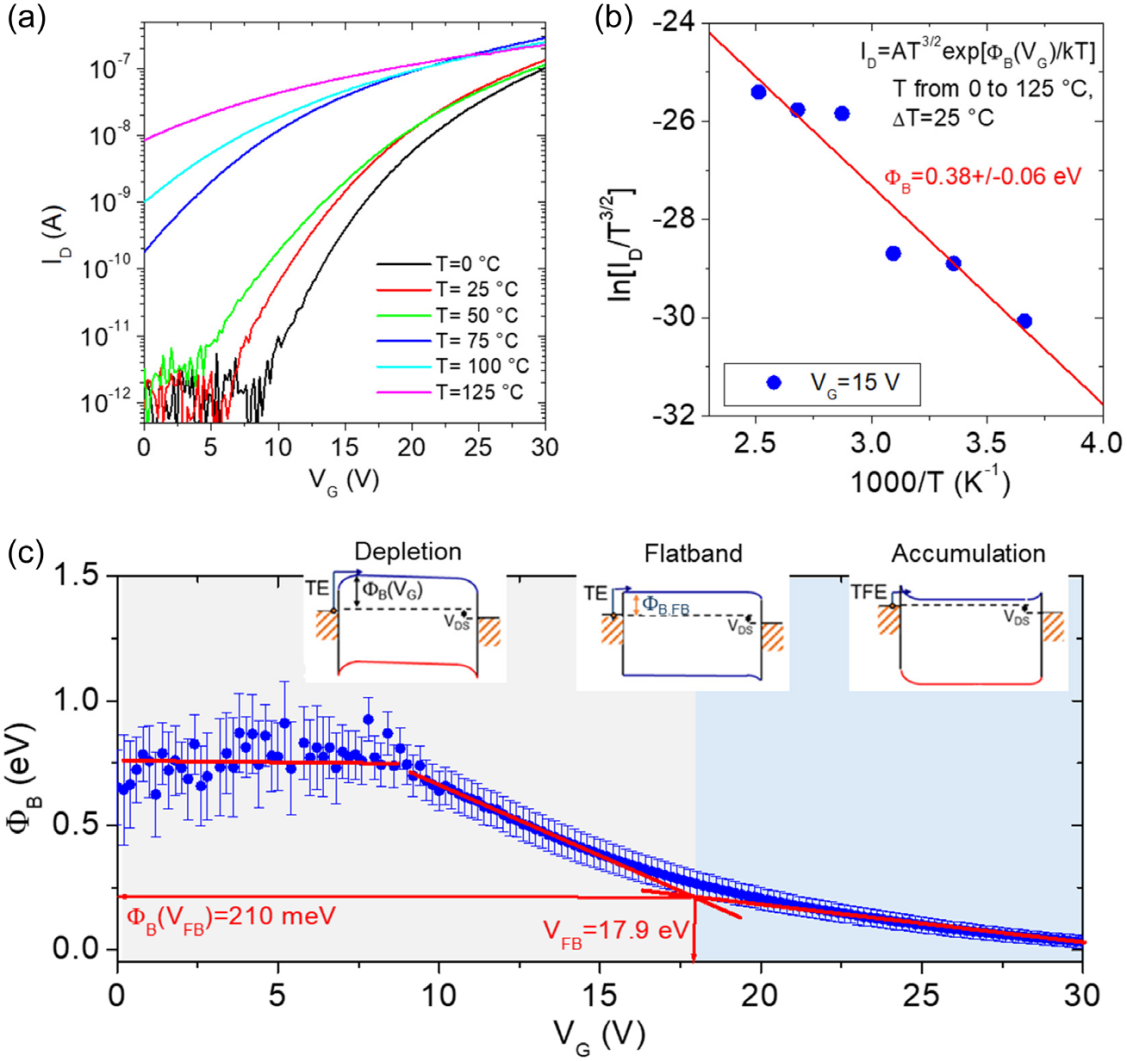
a) Subthreshold transfer characteristics *I*_D_–*V*_G_ of the 1L MoS_2_ FET at different temperatures from 0 to 125 °C. b) Arrhenius plot of ln[*I*_D_/T^3/2^] versus 1000/*T* at a fixed *V*_G_ = 15 V, from which an effective Schottky barrier Φ_B_ = 0.38 ± 0.06 eV is obtained by the slope of the linear fit. c) Gate bias dependence of the effective Φ_B_, and evaluation of the flatband voltage *V*_FB_ = 17.9 V and the corresponding effective Schottky barrier height Φ_B,FB_ = 210 meV. In the insets, illustrations of the current injection mechanisms under depletion, flatband, and accumulation conditions.

Transport properties of MoS_2_ FETs in the subthreshold regime are known to be dominated by the current injection mechanisms from source/drain contacts into the MoS_2_ channel.^[^ ^50^ ^]^ To investigate in detail the electrical properties of the Ni/Au contacts, particularly the Schottky barrier height Φ_B_ with 1L MoS_2_ channel, a temperature‐dependent analysis of the transfer characteristics has been carried out, as illustrated in **Figure** **5**. The strong increase of the subthreshold current *I*_D_ with increasing the *T* from 0 to 125 °C (Figure 5a) indicates the occurrence of a thermally activated current injection above the gate‐bias‐modulated Schottky barrier Φ_B_(*V*_G_), according to the relation

(2) $$I_{D}\approx T^{\frac{3}{2}}\exp\left[-\frac{\Phi_{B}(V_{G})}{k_{B}T}\right]$$

with the *T* ^3/2^ dependence in the pre‐exponential factor related to the 2D density of states in 1L MoS_2_.^[^ ^51^ ^]^ Figure 5b shows the Arrhenius plot of ln[*I*_D_/*T* ^3/2^] vs 1000/*T* (with *I*_D_ extracted from Figure 5a at a fixed *V*_G_ = 15 V), from which an effective Schottky barrier Φ_B_ = 0.38 ± 0.06 eV is obtained by the slope of the linear fit. By extending this procedure to all *I*_D_ values in the range of *V*_G_ from 0 to 30 V, the dependence of Φ_B_(*V*_G_) in the entire subthreshold regime is evaluated, as shown in Figure 5c. From this plot, the changes in the band‐bending of MoS_2_ channel and the corresponding current injection mechanisms as a function of *V*_G_ can be deduced, as depicted in the three energy band diagram schematics of Figure 5c. Specifically, in the flatband voltage condition (*V*_G_ = *V*_FB_), no band bending is present and the energy difference between the Fermi level and MoS_2_ conduction band corresponds to the “real” value of the Ni/MoS_2_ Schottky barrier (Φ_B,FB_). For *V*_G_ < *V*_FB_, an upward band bending occurs, corresponding to electron depletion from the channel, whereas *V*_G_ > *V*_FB_ results in a downward band bending, corresponding to electron accumulation. While thermionic emission (TE) above the Schottky barrier is the dominant current injection mechanism for *V*_G_ ≤ *V*_FB_, thermionic field emission (TFE) through the triangular barrier becomes dominant for *V*_G_ > *V*_FB_. The plot of experimental Φ_B_(*V*_G_) values (blue dots in Figure 5c) exhibits, in the depletion regime, a first part, from *V*_G_ = 0 to 7 V, with nearly constant Φ_B_ = 0.78 ± 0.14 eV, associated to a fully depleted 1L MoS_2_ channel, followed by a linear decrease of Φ_B_ as a function of *V*_G_, associated to the gradual reduction of MoS_2_ upward band bending. By linear fitting the experimental points in this region with the relation

(3) $$\Phi_{B}\left(V_{G}\right)=\Phi_{\text{B,FB}}-\gamma \left[V_{G}-V_{\text{FB}}\right]$$

the slope γ ≈ 60 meV V^−1^ is obtained, which is a measure of the band bending modulation efficiency by *V*_G_ in the depletion regime. The accumulation regime in Figure 5c is identified by a linear trend of Φ_B_ at high *V*_G_ values with a significantly smaller slope. Finally, the flat‐band voltage (*V*_FB_ = 17.9 V) and the corresponding effective Schottky barrier height value Φ_B,FB_ = 210 meV have been evaluated from the intersection of the linear fits in the two regimes, as illustrated in Figure 5c.

To better understand the MoS_2_ FET behavior, it is worth comparing the experimentally obtained *V*_FB_ value with the theoretical flat‐band voltage for a p^+^‐Si/SiO_2_/1L‐MoS_2_ metal‐insulator‐semiconductor (MIS) system, and the Φ_B,FB_ value with the ideal barrier for a Ni/MoS_2_ Schottky contact. Ideally, the *V*_FB_ value for the p^+^‐Si/SiO_2_/1L‐MoS_2_ system should be given by $V_{\text{FB,id}}=W_{p^{+}-\text{Si}}-W_{1L-{\text{Mos}}_{2}}\approx 0.66\;\text{eV}$, being $W_{p^{+}-\text{Si}}\approx \chi_{\text{si}}+E_{\text{g,Si}}=4.05+1.12=5.17\;\text{eV}$ the work‐function of the p^+^‐Si backgate, and $W_{1L-{\text{Mos}}_{2}}=\chi_{{\text{Mos}}_{2}}+\Phi_{\text{B,FB}}\approx 4.3+0.21=4.51\;\text{eV}$ the work‐function of *n*‐type doped 1L MoS_2_. The significantly higher experimental value of *V*_FB_ = 17.9 V can be accounted for by the presence of a density $N=\frac{C_{\text{ox}}\left(V_{\text{FB}}-V_{\text{FB,id}}\right)}{q}\approx 1.2\times 10^{12}{\;\text{cm}}^{-2}$ of negative charges in the SiO_2_ near‐interface region or on the surface of 1L MoS_2_. This charge is responsible for the normally off behavior of the transistor, that is, of the complete depletion of the MoS_2_ channel at *V*_G_ = 0 V. As previously discussed, these negative charges can be trapped within the different possible Na‐containing byproducts of LPI‐CVD reactions, including sodium molybdate (Na_2_MoO_4_), Na_2_O, Na—Mo—O—Si, and Na_2_SiO_3_.

According to the Schottky–Mott theory, the ideal value of the Ni/MoS_2_ Schottky barrier is given by $\Phi_{\text{B,id}}=W_{\text{Ni}}-\chi_{{\text{MoS}}_{2}}\approx 5.1-4.3=0.8\;\text{eV}$, much higher than the experimental value Φ_B,FB_ = 0.21 eV obtained from the temperature‐dependent analysis in Figure 5. Such a discrepancy from theoretical expectation is commonly ascribed to the Fermi level pinning effect at the metal/MoS_2_ interface, arising from damage formation or reactions in MoS_2_ during metal deposition.

In the following, spatially resolved electrical and optical spectroscopy analyses inside the channel region of the backgated MoS_2_ FET provided information on the lateral uniformity of doping and strain in the proximity of the source/drain contacts, which may affect the Schottky barrier and the overall electrical behavior of the device.

**Figure 6:**
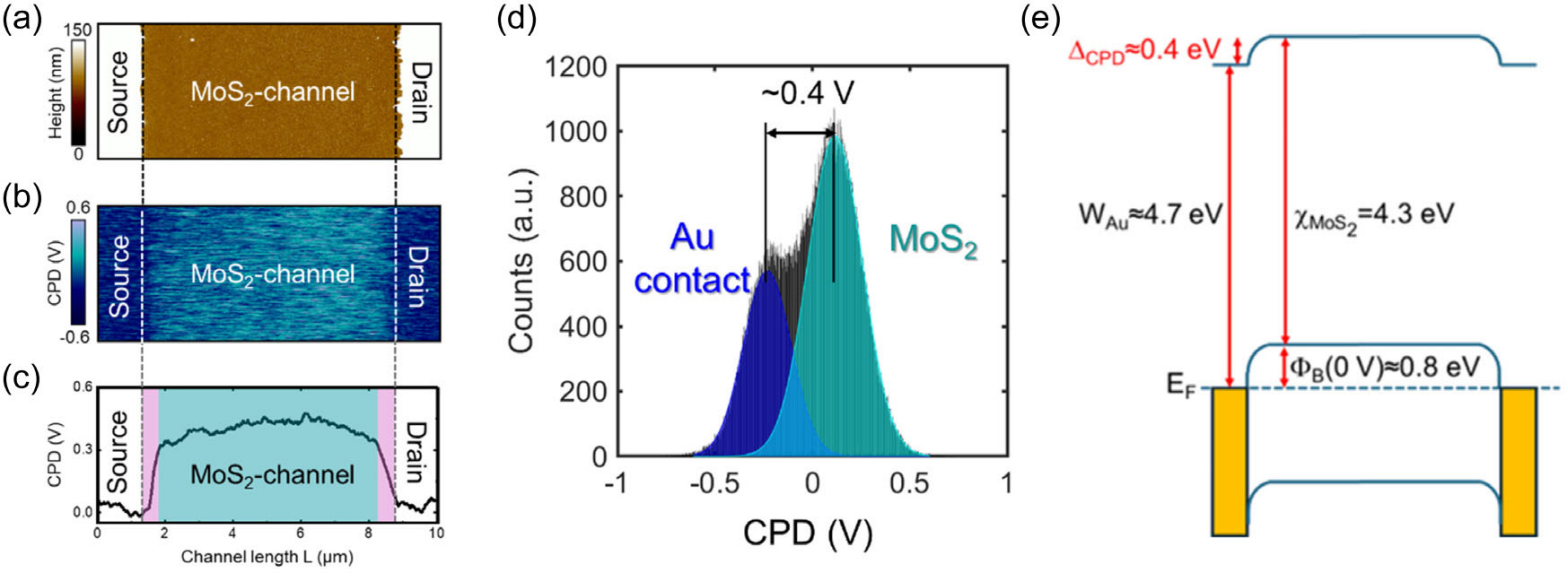
a) Morphological and b) CPD images acquired within the 1L MoS_2_ channel between the Ni/Au source/drain contacts in Peak Force KPFM mode. c) CPD profile extracted from the KPFM image, indicating a distinct difference between the Au surface and the fully depleted MoS_2_ channel. d) Distribution of the CPD values extracted from the KPFM image in (b), showing a Δ_CPD_ = CPD_MoS2_ − CPD_Au_ = 0.4 V between the channel and the contacts. e) Schematic energy band diagram of the channel, illustrating the correlation between Δ_CPD_ and the Schottky barrier height at *V*_G_ = 0 V from FET device characterization.

First, nanoscale resolution morphological and electrical measurements have been performed by KPFM on the back‐gated FET, with the sample placed on the grounded AFM chuck (i.e., at *V*_G_ = 0 V). Representative morphological and contact potential difference (CPD) images of a region including the MoS_2_ channel and the edge source and drain contacts are reported in **Figure** **6**a,b, respectively. The color map in Figure 6b shows an evident CPD contrast between the Ni/Au contacts (exposing Au surface) and MoS_2_, whereas smaller CPD variations are visible along MoS_2_ channel. The line profile along the channel length (Figure 6c) shows that the transition from the contact edge to MoS_2_ channel consists of a steep potential variation at the interface, followed by a smoother increase. To obtain a statistical evaluation of the surface potential distribution in the investigated area, Figure 6d reports the histogram of the CPD values extracted from Figure 6b. The two components associated with the Au and MoS_2_ surfaces can be clearly identified by Gaussian fitting of the histogram, and a variation of the CPD (Δ_CPD_≈0.4 V) between the two materials has been inferred from the peak's separation.

The results of this spatially resolved KPFM analysis provide a picture of the surface potential inside the channel at *V*_G_ = 0 V, that is, in the condition of a fully MoS_2_‐depleted channel, as demonstrated by electrical characterization of the macroscopic device in Figure 4 and 5 and, specifically, by the Φ_B_(*V*_G_) plot in Figure 5c. To correlate KPFM and device characterization results, a band diagram of the system is reported in Figure 6e, where the measured Δ_CPD_ ≈ 0.4 V from Figure 6d and the value of Φ_B_(0 V) ≈ 0.8 V from Figure 5c are reported, along with the electron affinity of 1L MoS_2_, $\chi_{{\text{Mos}}_{2}}\approx 4.3\;\text{eV}$. The work‐function value for the Au contacts, *W*_Au_ ≈ 4.7 eV, is consistent with recently reported values for Au(111) surfaces coated by adventitious carbon contamination after exposure to ambient conditions.^[^ ^52^ ^]^

**Figure 7:**
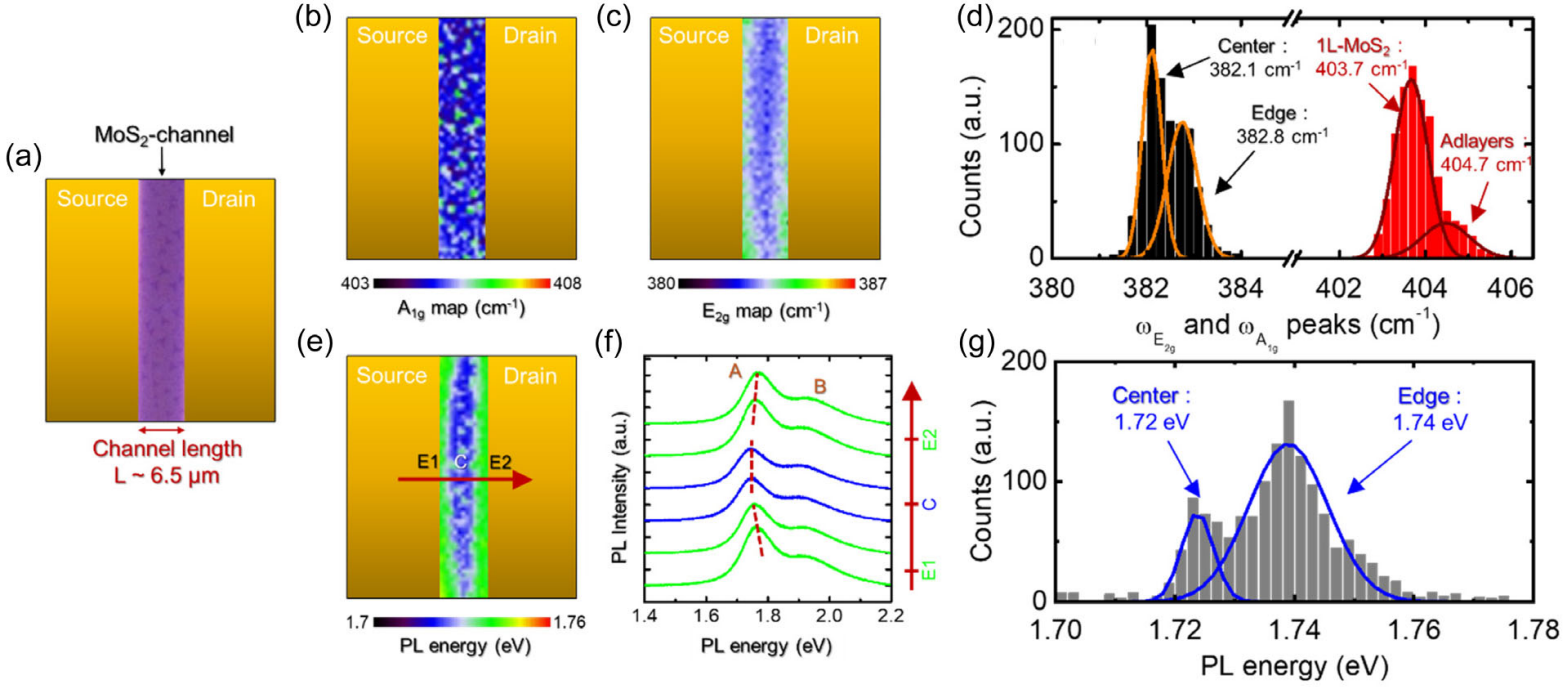
a) Optical image of the 1L MoS_2_ channel region between the source and drain contacts. Corresponding micro‐Raman maps of b) A_1g_ and c) E_2g_ peak frequencies inside the channel. d) Histograms of the E_2g_ (black bars) and A_1g_ (red bars) peak frequencies extracted from the maps in (b) and (c). Gaussian fitting of the E_2g_ distribution allows to identify two components, associated with tensile strain differences between the central and edge regions of the channel. Gaussian fitting of the A_1g_ distribution allows to separate a main component, associated with uniformly doped 1L MoS_2_, and a smaller contribution, associated with adlayers. e) Map of the PL peak energy inside the channel and f) representative PL spectra extracted along the channel, at positions indicated by the red arrow. g) Histograms of the PL peak energies extracted from the corresponding map, where the components associated with the central and edge region of the channel have been identified by Gaussian fitting.

After the nanoscale resolution KPFM‐based electrical characterization of the device, optical spectroscopy techniques, that is, micro‐Raman and micro‐PL spectroscopy, have been employed to extract information on the uniformity of strain and doping distribution within the 1L MoS_2_ channel. The lateral resolution of these techniques, corresponding to the spot size of the excitation laser (wavelength *λ* = 532 nm) focused with a 100× objective and approximately equal to 1 μm, is enough to provide spatially resolved analyses within a channel length *L* = 6 μm. **Figure** **7**a shows an optical image of the channel region between source and drain contacts, where the violet contrast indicates a uniform 1L MoS_2_ coverage on SiO_2_, with small triangular darker regions associated with a few isolated MoS_2_ adlayers nucleated on 1L MoS_2_ surface during LPI‐CVD. Figure 7b,c shows the corresponding color scale maps of the frequencies for the out‐of‐plane (A_1g_) and in‐plane (E_2g_) Raman modes of MoS_2_. While the A_1g_ frequency distribution is nearly uniform inside the channel, with small fluctuations associated with the isolated triangular adlayers observed in the optical image, the E_2g_ frequency map exhibits clear differences between the central region and the edge regions of the channel close to source/drain contacts. The different uniformity of the A_1g_ and E_2g_ distributions can also be deduced by looking at the histograms of the A_1g_ and E_2g_ values (Figure 7d) extracted from the maps. While the A_1g_ distribution (red histogram) could be fitted by a main Gaussian contribution peaked at 403.7 cm^−1^, associated with 1L MoS_2_, and a very small contribution (peak at 404.7 cm^−1^) associated with triangular adlayers, in the case of E_2g_ distribution (black histogram), two Gaussian contributions with similar weights can be identified by the fit, associated with the central region (peak at 382.1 cm^−1^) and the edge region (peak at 382.8 cm^−1^), respectively, of the channel. Since the E_2g_ mode is mainly sensitive to MoS_2_ strain variations, with a red‐shift of this peak associated with increased compressive strain (or, alternatively, reduced tensile strain), the E_2g_ map in Figure 7c clearly indicates the presence of an inhomogeneous strain field along the channel length direction. To get further insight into this observation, a spatially resolved PL spectroscopy analysis of the same device channel areas was also carried out. Noteworthy, the obtained color map of PL peak energy (Figure 7e) is in striking agreement with the E_2g_ map, showing a blue‐shift of the PL energy near the contact edges as compared to the channel center. Representative PL spectra, reported in Figure 7f, have been extracted along the channel length direction indicated by the red arrow in Figure 7e. Moving from the MoS_2_ channel edges close to the source and drain contacts (positions E1 and E2) to the center of the channel (position C), the main exciton peak A underwent an evident red‐shift (from the green to the blue spectra). To get a deeper insight into the observed changes, a deconvolution analysis with two excitonic (A^0^, B) and a trionic (A^−^) component of two representative PL spectra collected at the edge and at the center of the channel is also reported in Figure S4, Supporting Information. A collective blue‐shift of 20 meV for all the components was deduced when moving from the center to the edges.

Finally, Figure 7g shows the histogram of PL energies extracted from the map in Figure 7e, where two main contributions can be identified at 1.72 eV and at 1.74 eV, associated with the central region and edges of the MoS_2_ channel, respectively. In particular, the blue‐shift of PL energy distribution can be ascribed to a reduced tensile strain in the edge region of the channel near the source and drain contacts.^[^ ^53^, ^54^, ^55^, ^56^ ^]^ Thus, the deposition of Ni/Au source contacts has a significant impact on both the vibrational and optical properties of the MoS_2_ system.

**Figure 8:**
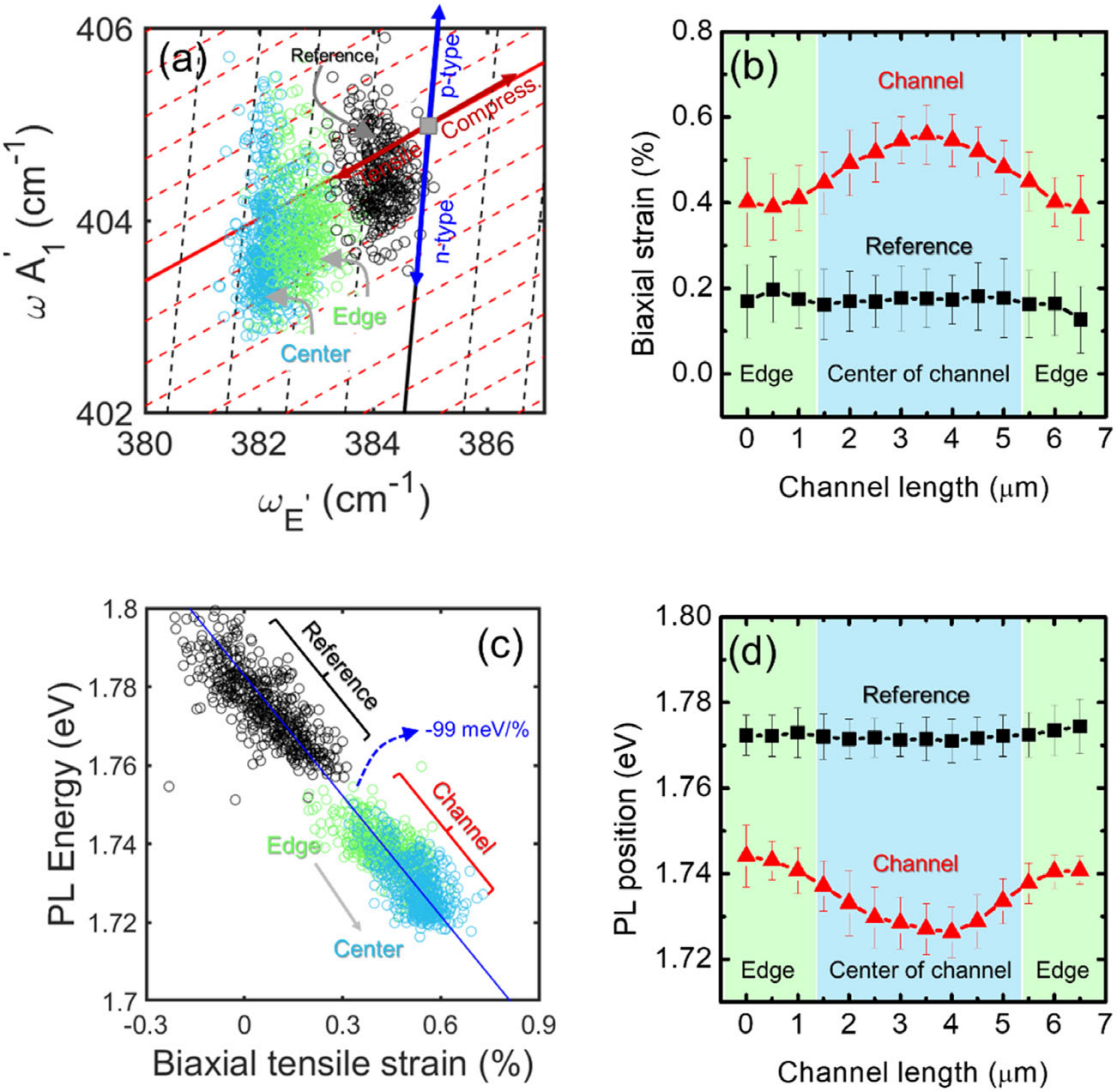
a) Correlative E_2g_ versus A_1g_ plot for the strain and doping evaluation on as‐grown 1L MoS_2_ (black circles) and inside the channel of a backgated 1L MoS_2_ FET. Here, data points from the edges and the center of the MoS_2_ channel are indicated by green and cyan circles, respectively. b) Biaxial tensile strain distribution as a function of the position within the channel length *L* and average value of the strain for as‐deposited MoS_2_, taken as reference. c) PL peak energy as a function of the biaxial strain calculated from Raman measurements on the as‐deposited MoS_2_ and within the channel of the FET. The behavior of the three clouds of data‐points in good agreement with the literature linear dependence of ≈−99 meV/%. d) PL peak energy as a function of L, showing a ≈20 meV blue‐shift from the center to the edges. The average PL peak energy measure on as‐deposited 1L MoS_2_ is reported as a reference.

To quantitatively evaluate the strain distribution within the 1L MoS_2_ channel, a correlative plot of the $\omega_{A_{1g}}$ versus $\omega_{E_{2g}}$ data points extracted from Figure 7b,c has been built, as displayed in **Figure** **8**a. For clarity, the data points extracted from the central and edge regions of the channel are represented by cyan and green circles, respectively. Furthermore, the black circles represent the data points extracted from Raman maps acquired on a representative as‐grown 1L MoS_2_ flake before processing for contact fabrication (see Figure S1a,b, Supporting Information).

In this plot, the red continuous line represents the “strain‐line”, that is, the theoretical $\omega_{A_{1g}}$ versus $\omega_{E_{2g}}$dependence for ideally undoped 1L MoS_2_ subjected only to strain, whereas the black continuous line is the “doping‐line”, that is, the theoretical dependence for ideally unstrained 1L MoS_2_ subjected only to doping. The gray square at the crossing point between the two lines represents the ideal case of unstrained and undoped 1L MoS_2_. The experimental values measured on a suspended 1L MoS_2_ membrane ($\omega_{E_{2g}}^{0}=385\;{\text{cm}}^{-1}$, $\omega_{A_{1g}}^{0}=405{\text{ cm}}^{-1}$) have been taken from the literature as the best approximation for the ideal unstrained and undoped material.^[^ ^57^ ^]^ The strain and doping lines separate the diagram in Figure 8a into four regions with different combinations of tensile/compressive strain and *p*/*n* type doping, as indicated by the red and blue arrows. Furthermore, the dashed black and red lines serve as guides for the eye to evaluate the range of strain and doping covered by the experimental data points. According to this plot, the data points of the as‐grown flake span a doping range from ≈−2 × 10^12^ cm^−2^ (*n*‐type) to ≈2 × 10^12^ cm^−2^ (*p*‐type), and those in the FET channel span a range from ≈−3 × 10^12^ cm^−2^ (*n*‐type) to ≈3 × 10^12^ cm^−2^ (*p*‐type), independently of the region (edge and center). These results are in agreement with those from FET electrical characterization and KPFM mapping (at *V*_G_ = 0 V), indicating a depleted channel (i.e., net carrier density *n* ≈ 0) with nearly uniform potential distribution along the channel length *L*. On the other hand, an overall increase in the biaxial tensile strain can be observed moving from the cloud of data points acquired on the as‐grown flake to that acquired in the FET channel. Furthermore, a clear decrease of the average tensile strain values from the central to the edge region of the channel can be deduced from this latter data point distribution.

The geometrical approach illustrated in Figure 8a allows for converting each ($\omega_{A_{1g}}$,$\omega_{E_{2g}}$) experimental data point into the corresponding strain (*ε*) and doping (*n*) values by solving the following system of equations^[^ ^58^ ^]^

(4a) $$\omega_{E_{2g}}=\omega_{E_{2g}}^{0}-2\gamma_{E_{2g}}\omega_{E_{2g}}^{0}\varepsilon +k_{E_{2g}}n$$

(4b) $$\omega_{A_{1g}}=\omega_{A_{1g}}^{0}-2\gamma_{A_{1g}}\omega_{A_{1g}}^{0}\varepsilon +k_{A_{1g}}n$$

where $\gamma_{E_{2g}}=0.68$ and $\gamma_{A_{1g}}=0.21$ are the Grüneisen parameters for 1L MoS_2_,^[^ ^57^ ^]^ while $k_{E2g}=-0.33\times 10^{‐13}\text{cm}$ and $k_{A1g}=-2.2\times 10^{‐13}\;\text{cm}$ are the shift rates of the Raman peaks as a function of the electron density (*n*).^[^ ^59^ ^]^ By using these equations, the $\omega_{A_{1g}}$ and $\omega_{E_{2g}}$ maps in Figure 7b,c were converted into maps of strain and doping distribution, as reported in Figure S5a,b, Supporting Information. Figure 8b, red triangles, shows the evaluated trend of the biaxial strain *ε* along the channel length *L*, where each data point and the error bar are the average and the standard deviation along the channel width *W*. The observed decrease from *ε* = (0.56 ± 0.07)% to *ε* = (0.40 ± 0.08)% moving from the central region to the edges near the source and drain contacts can be ascribed to a compressive effect of the deposited Ni/Au contacts, partially compensating the tensile strain of the 1L MoS_2_ membrane associated with the interaction with the SiO_2_ substrate. As a reference, the constant strain value inside an as‐grown flake is reported by black squares.

To get a deeper insight into the correlation between biaxial strain and PL emission in the channel, Figure 8c displays a plot of the PL energy versus biaxial strain distributions extracted from the maps in Figure 7b and Figure S1d, Supporting Information. The cloud of data points exhibits an elongated shape, with a clear red‐shift of PL emission with increasing the strain from the edge (green circles) to the center (cyan circles) of the channel. Noteworthy, the slope of this distribution (≈−99 meV/%) is in excellent agreement with previous experimental and theoretical results of the PL energy shift rate as a function of strain,^[^ ^57^ ^]^ confirming that the observed changes in the PL emission are due to strain‐induced energy band‐structure modifications of 1L MoS_2_. Finally, a plot of the PL energy versus position along the channel length is reported in Figure 8d, showing a decrease from ≈1.74 eV at the edges to a minimum value of ≈1.72 eV at the center of the channel, corresponding to the position of the maximum in the biaxial strain profile of Figure 8b.

**Figure 9:**
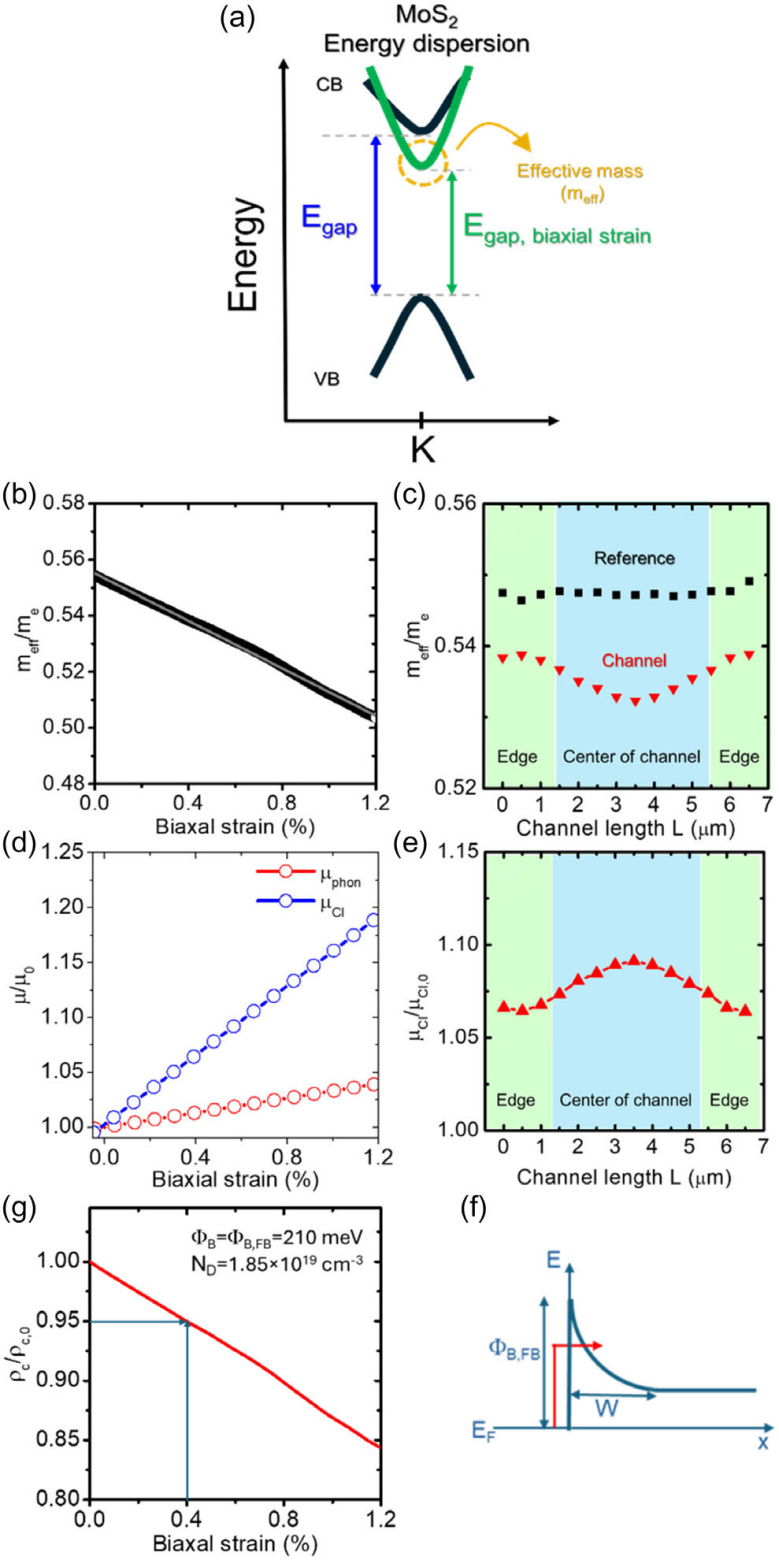
a) Schematic illustration of the band‐structure modification for tensile‐strained 1L MoS_2_ (green bands) as compared to unstrained films (blue bands). b) Theoretical dependence of the *m*_eff_ on the biaxial tensile strain by DFT calculations. c) Evaluated *m*_eff_/*m*_e_ distribution along the channel length *L* from the biaxial strain profile (red triangles), compared with the *m*_eff_/*m*_e_ distribution in a pristine MoS_2_ flake, used as a reference (blue squares). d) Calculated biaxial strain dependence of the mobility limited by charged impurity scattering (*μ*_CI_) and by intrinsic phonon scattering (*μ*_phon_), both normalized to the corresponding mobility values *μ*_CI,0_ and *μ*_phon,0_ at *ε* = 0. e) Evaluated *μ*_CI_/*μ*_CI,0_ distribution along the channel length *L*. f) Schematic of current injection through the contact/1L MoS_2_ triangular barrier in the FET under accumulation conditions, and g) calculated dependence of the specific contact resistance (*ρ*_C_/*ρ*_C,0_) on the biaxial strain according to the TFE model for Φ_B_ = Φ_B,FB_ = 210 meV and *N*_D_ = 1.85 × 10^19^ cm^−3^. The arrows indicate the value of *ρ*_C_/*ρ*_C,0_ corresponding to the local strain (≈0.4%) of 1L MoS_2_ close to the source/drain contacts. Data reported in panels (b) and (d) extracted from ref. [60].

The observed variations of the strain distribution along the channel length L have significant implications for transport phenomena in 1L MoS_2_ FETs. In fact, uniaxial and biaxial tensile strains of ultra‐thin MoS_2_ films result in the modification of 1L MoS_2_ band structure, particularly in a narrowing of the direct bandgap and a change in the curvature of the conduction band minimum at the K point of the 1st Brillouin zone (see schematic in **Figure** **9**a). This latter translates into a reduction of the electron's effective mass (*m*_eff_ = *ħ* ^2^/(ꝺ^2^ *E*/ꝺ*k* ^2^)) with increasing the tensile strain, as shown in Figure 9b, reporting literature results of DFT calculations on 1L MoS_2_ for the typical range of biaxial strain accessed experimentally.^[^ ^60^ ^]^ In our 1L MoS_2_ FET, the strain decrease from the center to the edges of the channel gives rise to a relatively small increase of effective mass, as shown in Figure 9c, and in the energy bandgap (≈20 meV), as demonstrated in Figure 8d.

Since *m*_eff_ is a key physical parameter for the main carrier scattering mechanisms in 2D semiconductors, the reduction of m_eff_ in tensile‐strained 1L MoS_2_ (for the considered range of biaxial strain values) is expected to translate into an improvement of the electron mobility. Figure 9d shows the biaxial strain dependences (extracted from recently reported DFT calculations)^[^ ^60^ ^]^ of the mobility limited by charged impurity scattering (*μ*_CI_/*μ*_CI,0_) and by intrinsic phonon scattering (*μ*_phon_/*μ*_phon,0_), both normalized to the corresponding mobility values *μ*_CI,0_ and *μ*_phon,0_ at *ε* = 0. According to these calculations, both *μ*_CI_/*μ*_CI,0_ and *μ*_phon_/*μ*_phon,0_ exhibit an increase with the biaxial strain (i.e., with the reduction of *m*_eff_), although the improvement of charged‐impurity limited mobility is more significant (up to 20% in the considered strain range). As a matter of fact, this mechanism is expected to play a major role in the electronic transport of our backgated 1L MoS_2_ FETs without high‐*k* encapsulation. Hence, in Figure 9e, we reported the *μ*_CI_/*μ*_CI,0_ distribution along the channel length L, evaluated according to the dependence in Figure 9d from the biaxial strain distribution in Figure 8b and, consequently, the effective mass distribution in Figure 9c. This plot indicates that the local mobility in the central region of the channel exhibits a moderate improvement, as compared to the regions close to the contacts.

Furthermore, the local *m*_eff_ value in the MoS_2_ region at source/drain edges is expected to have an impact on the FET contact resistance in the accumulation regime, where current injection is dominated by TFE, as depicted in Figure 9f. In fact, the specific contact resistance *ρ*_c_ in the TFE regime depends on the metal/MoS_2_ Schottky barrier Φ_B_, on the effective mass and doping *N*_D_ as

(5) $$\rho_{\text{C,TFE}}\propto \exp\left[\frac{\Phi_{B}}{E_{0}}\right]$$

where $E_{0}=E_{00}\coth\left(\frac{E_{00}}{kT}\right)$ and $E_{00}=\frac{qh}{4\pi }\sqrt{\frac{N_{D}}{\varepsilon_{0}\varepsilon_{\text{MoS}2}m_{\text{eff}}}}$, being h the Planck's constant, and *ε*_MoS2_ = 4.6 the permittivity of 1L MoS_2_. Figure 9g reports the calculated dependence of *ρ*_c_/*ρ*_c,0_ on the biaxial strain (according to Equation (5)) for the metal/MoS_2_ Schottky barrier Φ_B_ =Φ _B,FB_ = 210 meV and a doping *N*_D_ = *N*/*t* = 1.85 × 10^19^ cm^−3^, being *N* = 1.2 × 10^12^ cm^−2^ the charge density at the interface with SiO_2_ and *t* = 0.65 nm the nominal thickness of 1L MoS_2_. A reduction of the specific contact resistance is predicted with increasing the biaxial strain (i.e., decreasing *m*_eff_) of 1L MoS_2_ at the interface with the contacts. In particular, the local biaxial strain of ≈0.4% (i.e., a local *m*_eff_ ≈ 0.54 *m*_e_) of 1L MoS_2_ at the Ni/Au contact's interface corresponds to *ρ*_c_ ≈ 0.95 *ρ*_c,0_ lower than the unstrained value *ρ*_c,0_, as indicated by the arrows in Figure 9g.

Although the measured variations of the strain distribution along the channel length have a moderate effect on the mobility distribution and current injection in our test devices with micrometer channel length, it is expected that accounting for actual MoS_2_ strain and bandgap variations inside the channel will be more critical in the case of ultra‐scaled MoS_2_ transistors with nanoscale channel length.

## 3 Conclusion

In conclusion, we reported a multiscale investigation of large area 1L MoS_2_ domains grown by LPI‐CVD on SiO_2_/Si substrates, starting from the compositional, structural, and vibrational/optical analysis of as‐deposited material up to the comprehensive characterization of backgated FETs. The devices exhibit very attractive properties for ultra‐low power applications, such as an *I*_on_/*I*_off_ > 10^6^ and a normally‐ off electrical behavior, with the positive *V*_th_ (i.e., fully depleted channel at *V*_G_ = 0 V) ascribed to negative charges in the near interface region of SiO_2_. The FET behavior in the subthreshold regime was dominated by the gate bias‐dependent Schottky barrier between Ni/Au source/drain contacts and MoS_2_ channel, with a Φ_B,FB_ = 0.21 eV at the flatband voltage *V*_FB_ = 17.9 V evaluated by temperature‐dependent *I*_D_–*V*_G_ analyses, consistently with spatially resolved surface potential mapping of the channel by KPFM. The combination of micro‐Raman and micro‐PL mapping revealed an overall increase in the average biaxial tensile strain (*ε*) and a red shift of PL energy distribution starting from as‐deposited 1L MoS_2_ domains to samples with fabricated devices, which was ascribed to device processing steps. Interestingly, inhomogeneous strain and PL energy distribution were observed within the FET channel, with a reduced *ε* and blue‐shifted PL energy close to the Ni/Au source/drain contacts, suggesting a biaxial compression of 1L MoS_2_ induced by metal deposition, which partially compensates the average tensile strain inside the channel. Finally, we evaluated the impact of the measured strain variations along the channel length on the distribution of the effective mass, and its consequent effects on the carrier mobility distribution and on the specific contact resistances at source/drain contacts. Although the strain variations in our micrometer channel devices have only a moderate effect on the local mobility and current injection, it is expected that these effects will be extremely relevant in ultra‐scaled MoS_2_ transistors with complex geometries, such as FinFETs or multilevel circuits. Hence, the multiscale characterization methodology demonstrated in this article will represent a powerful tool for a complete description of the electrical behavior and modeling of these advanced devices.

## 4 Experimental Section

4.1

4.1.1

##### Sample Growth

Molybdenum precursor consisted of an aqueous mixture containing the solution of ammonium heptamolybdate tetrahydrate (AHT, Sigma–Aldrich, 99.98%) 0.003 mol L^−1^ and the promoter solution (NaOH) 0.060 mol L^−1^. Indeed, OptiPrep (a solution of 60% w/v iodixanol in water—CAS Number 92339–11‐2) was employed as a density gradient with a concentration of 0.5 for 5 mL of solution. 30 μL of solution was spin coated on SiO_2_/Si substrate at 3000 rpm for 30 s.

The sulfurization process has been carried out inside an open quartz tube (1 m long and with a diameter of 4 cm) with two separated heating zones. The first was used for the evaporation of the sulfur powder (300 mg, Sigma–Aldrich, 99.98%) located in a quartz boat at 160 °C, while the second was used for the MoS_2_ growth on the substrate at 820 °C. The entire process was achieved at atmospheric pressure and employing a continuous nitrogen gas flux at 200 sccm.

##### Back‐Gated Field‐Effect Transistor Fabrication

The pattern design for source and drain contacts has been carried out employing a direct laser writing lithographic system, model DWL 66+ by Heidelberg Instruments. After the development of the structures, the metallic contacts have been fabricated by e‐beam evaporation of Ni (20 nm) and Au (80 nm) under high vacuum (base vacuum 10^−7^ mbar) using an EvoVac physical vapor deposition (PVD) system by Ǻngstrom Engineering.

##### XPS and XAS Characterizations

The XPS and X‐ray absorption near‐edge structure (XANES) spectroscopy were carried out at the BACH beamline of CNR at the Elettra synchrotron radiation facility (Trieste, Italy). Photoelectron spectra were collected in normal emission geometry at a take‐off angle of 90° using a Scienta R3000 hemispherical analyzer at an angle of 60° with respect to the incident beam direction. The X‐rays were linearly polarized with the polarization vector parallel to the scattering plane and the beam size on the sample was ≈500 × 300 μm^2^. The MoS_2_ layers and the SiO_2_ substrate showed differential surface charging under the X‐ray irradiation. The binding energies of the MoS_2_ spectra were referenced to the spectra of a MoS_2_ powder sample (Acros Organics, 98.5%) mounted on a conductive carbon tape, while other spectra were referenced to the Si 2*p* peak of SiO_2_ (103.5 eV). Beam‐induced damage was continuously monitored and minimized by moving the beam across the sample surface. Photoemission spectra were fitted using Voigt line shapes and a Shirley or linear type background. The total instrumental energy resolution was 0.2 and 0.6 eV at a photon energy of 600 and 1250 eV, respectively.

Na K‐edge XANES spectra were acquired in total electron yield (TEY) mode at an incidence angle of 30° relative to the sample surface. The energy resolution was set to 0.2 eV, and the signal intensities were normalized to the incoming beam flux. The photon energy scale was calibrated by measuring the Au 4*f* signal from a gold foil.

##### AFM, KPFM and SEM Characterization

Morphological, phase analysis, and step height evaluation on 1L MoS_2_ deposited on SiO_2_/Si have been carried out employing a Bruker Icon Dimension system in PeakForce tapping mode with triangular tips (model PFQNE‐AL by Bruker) featured by a nominal curvature radius of ≈5 nm and an oscillation frequency of 300 kHz. Same tips have been employed in PeakForce KPFM mode for the evaluation of the contact potential difference between the MoS_2_ and the metallic contacts. Evaluation of the triangular MoS_2_ flakes on a larger area was achieved by SEM characterization employing a Zeiss Auriga Compact system equipped with a GEMINI Field‐Emission column.

##### Electrical Characterizations

Temperature‐dependent current‐voltage (*I*–*V*–*T*) characterization of the backgated 1L MoS_2_ FETs has been carried out using a Keysight B1500 A parameter analyzer and its medium power modules in an MPI TS 200 HP probe station.

##### Vibrational and Optical Characterizations

Micro‐Raman and micro‐PL measurements were carried out employing both a Renishaw InVia and a LabRam HR‐Evolution Spectrometer system (HORIBA France SAS, Lyon, France) equipped with 100× objectives. For Raman single spectrum and mapping, a grating of 1800 L mm^−1^ was employed. Differently, a grating of 1200 L mm^−1^ was used for the PL characterization.

## Supporting Information

Supporting Information is available from the Wiley Online Library or from the author.

## Conflict of Interest

The authors declare no conflict of interest.

## Supporting information

Supplementary Material

## Data Availability

The data that support the findings of this study are available from the corresponding author upon reasonable request.
