# Supplementary material for: Impact of the Schottky Barrier and Contact‐Induced Strain Variations inside the Channel on the Electrical Behavior of Monolayer MoS2 Transistors
Source: Small Sci. 2025 Sep 28;5(12):e202500244. doi: 10.1002/smsc.202500244 (PMC12697886; doi:10.1002/smsc.202500244)
Supplement: Supplementary file 1 — Supplementary Material [file SMSC-5-e202500244-s001.pdf]

Supporting Information

Impact of the Schottky barrier and contact induced strain variations along the channel on MoS_2 transistors electrical behaviour

*S.E. Panasci [1], E. Schilirò [1], G. Greco [1], P. Fiorenza [1], M. Vivona [1], S. Di Franco [1], F. Roccaforte [1], F. Esposito [2,3], M. Bosi [2], G. Attolini [2], I. Píš [3], F. Bondino [3], M. Pedio[3], A. Madonia [4], M. Cannas [4], S. Agnello [4], L. Seravalli [2,**], F. Giannazzo [1,*]*

Dr. S.E. Panasci, Dr. E. Schilirò, Dr. G. Greco, Dr. P. Fiorenza, Dr. M. Vivona, Dr. S. Di Franco, Dr. F. Roccaforte, Dr. F. Giannazzo
Consiglio Nazionale delle Ricerche, Istituto per la Microelettronica e Microsistemi (CNR-IMM), Strada VIII, 5, Zona Industriale, I-95121 Catania, Italy
*e-mail: filippo.giannazzo@imm.cnr.it

F. Esposito, Dr. M. Bosi, Dr. G. Attolini, Dr. L. Seravalli
Consiglio Nazionale delle Ricerche, Istituto dei Materiali per l'Elettronica ed il Magnetismo (CNR-IMEM), Parco Area delle Scienze 37/a, 43124 Parma, Italy
**e-mail: luca.seravalli@imem.cnr.it

Dr. I. Píš, Dr. F. Bondino, Dr. M. Pedio
Consiglio Nazionale delle Ricerche (CNR) - Istituto Officina dei Materiali (IOM), Area Science Park, S.S. 14 Km. 163, 5, Basovizza, I-34149 Trieste, Italy

Dr. A. Madonia, Prof. M. Cannas, Prof. S. Agnello
Department of Physics and Chemistry Emilio Segré, University of Palermo, Via Archirafi 36, 90143 Palermo, Italy

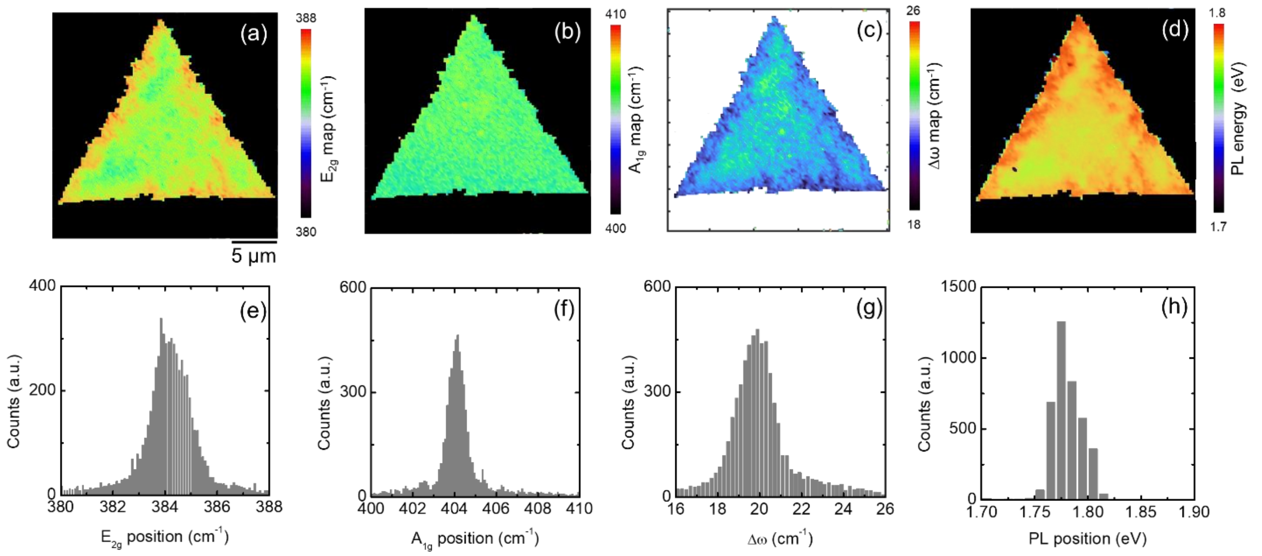

Figure S1.- Raman mapping carried out to extrapolate on the as-deposited 1L MoS_2: (a) the E_{2g}, (b) A_{1g} and (c) Δω variations. PL energy map on the as-deposited 1L MoS_2. Corresponding histogram of the previous Raman and PL maps for the (e) E_{2g}, (f) A_{1g}, (g) Δω and (h) PL variations, respectively.

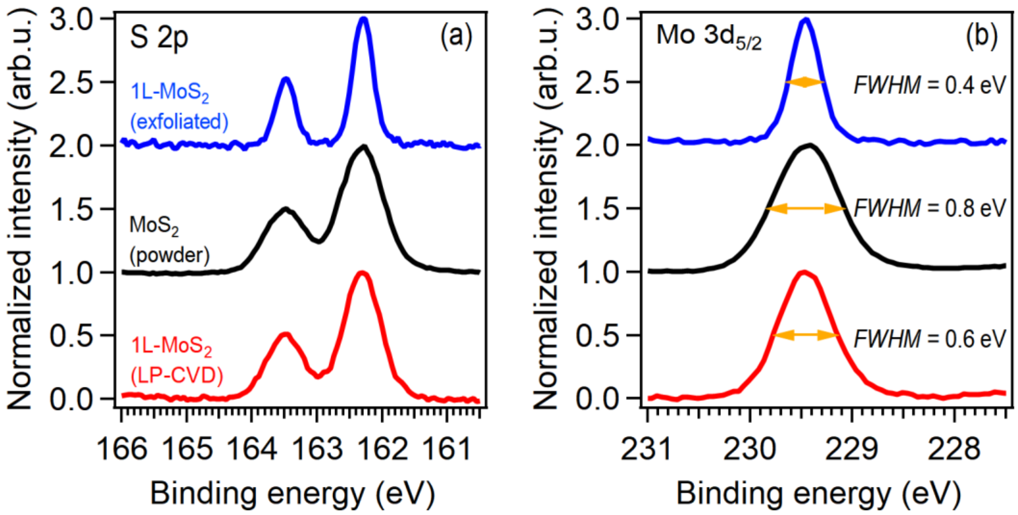

Figure S2.- S 2p and Mo $3d_{5/2}$ XPS spectra of 1L MoS_2 flakes grown on a SiO_2/Si substrate by LP-CVD (red curves), compared to highly crystalline 1L MoS_2 exfoliated from a bulk MoS_2 crystal and subsequently transferred to a SiO_2/Si substrate (blue curves). XPS spectra of a reference MoS_2 powder sample (Acros Organics, 98.5%) are also included for comparison (black curves). The sharper peaks indicate a higher crystalline quality.

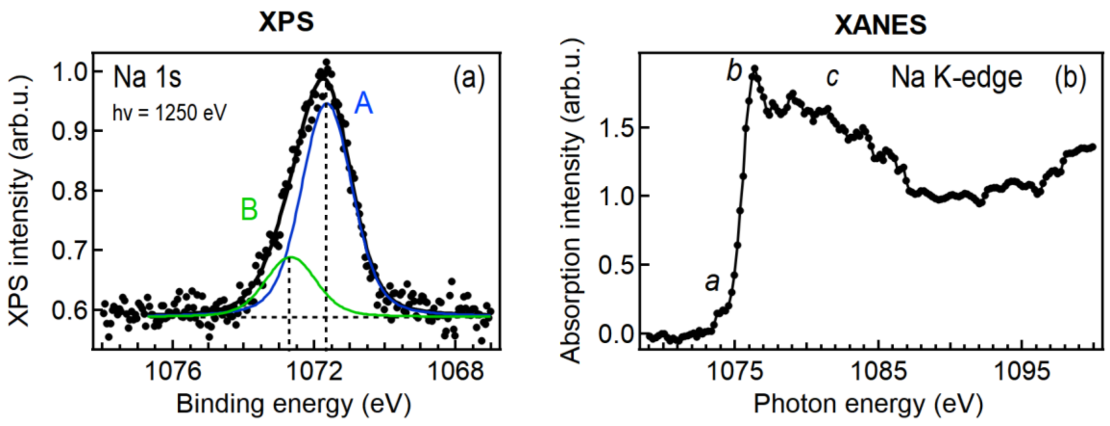

Figure S3.- Na 1s XPS and Na K-edge XANES spectra recorded after the synthesis of 1L-MoS_2 on a SiO_2/Si substrate using liquid-precursor CVD with NaOH as a promoter.

The Na 1s signal observed in XPS after synthesis of MoS_2 from the liquid precursor with the NaOH promoter is shown in **Figure S3**(a). The peak shape indicates the presence of Na in several chemical states at or near the surface. The main component A, at a binding energy of ~1071.7, eV could originate from the possible residues of sodium molybdate (Na_2MoO_4) and Na-Mo-O-Si intermediate compounds formed during CVD growth [1]. However, the molar ratio Na:Mo ~ 9, estimated from the XPS peak intensities and the corresponding relative sensitivity factors, suggests that other Na-containing compounds also contribute to this signal. One of these could be Na_2SiO_3 [2], however, the measured Na K-edge XANES spectrum (**Figure S3b**) does not match the reported absorption spectra for this compound [3]. While the presence of Na_2SiO_3 is not ruled out by the XANES spectrum, it is probably not the dominant sodium-containing phase. Another possible by-product formed during the conversion of sodium molybdate intermediate to MoS_2 is sodium oxide (Na_2O), which is assumed to be present in the form of small clusters at the MoS_2 edges and which can migrate to the SiO_2 substrate and form sodium silicate glasses at high temperatures. The presence of the second Na 1s component at the higher binding energies (B) is consistent with sodium diffusion into the silica substrate [4].

The Na K-edge XANES spectrum (**Figure S3** (b)), collected in total electron yield mode, displays a weak pre-edge peak *a* at 1074 eV, which is assigned to 1s → 3s transitions formally forbidden in

purely octahedral environment [5,6], a peak *b* at 1076.3 eV, and a broad feature *c* around 1082 eV, which are associated with electron transitions from the 1s core level to the 3p states of Na^+ [5,6]. No distinct features are observed above 1090 eV. The presence of broad features is typical of Na in disordered environments, such as those found in glassy silicate compounds [5,6,7]. The measured Na K-edge spectrum approximately resembles spectra reported for Na_2SO_4 [8], however, sulfate and sulfite signals are not clearly detected in the S 2p XPS spectrum, indicating that the presence of this compound on the surface is negligible.

In conclusion, the absence of sharp peaks in the sodium XANES spectrum suggests that the formed Na compounds are disordered, glassy, or amorphous compounds composed of various Na sites and phases with a broad distribution of Na–O distances.

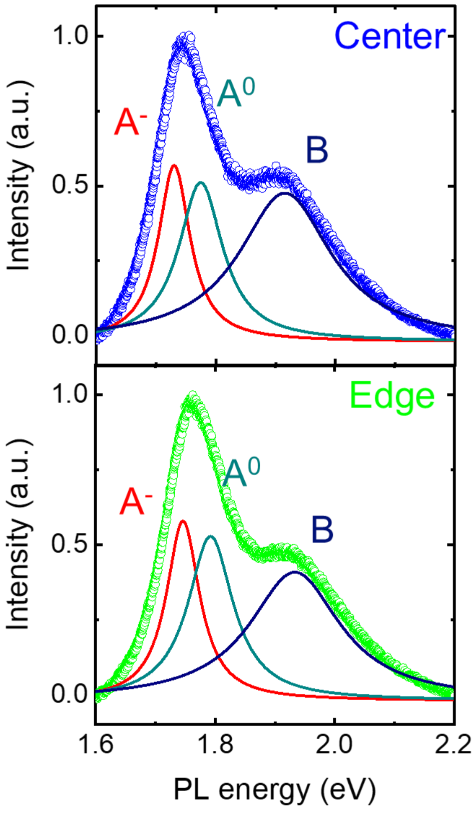

Channel position	Trion A^-	Exciton A^0	Exciton B
Edge	1.75 eV	1.79 eV	1.93 eV
Center	1.73 eV	1.77 eV	1.91 eV

Figure S4.- Comparison of two representative PL spectra extracted from the centre (blue spectrum) and the edge (green spectrum) of the MoS_2 channel. After a deconvolution analysis, the three main components (A^-, A^0 and B) have been indicated and evaluated in the corresponding table, showing a

collective shift of the contributions passing from the edge near the contact to the centre of the channel.

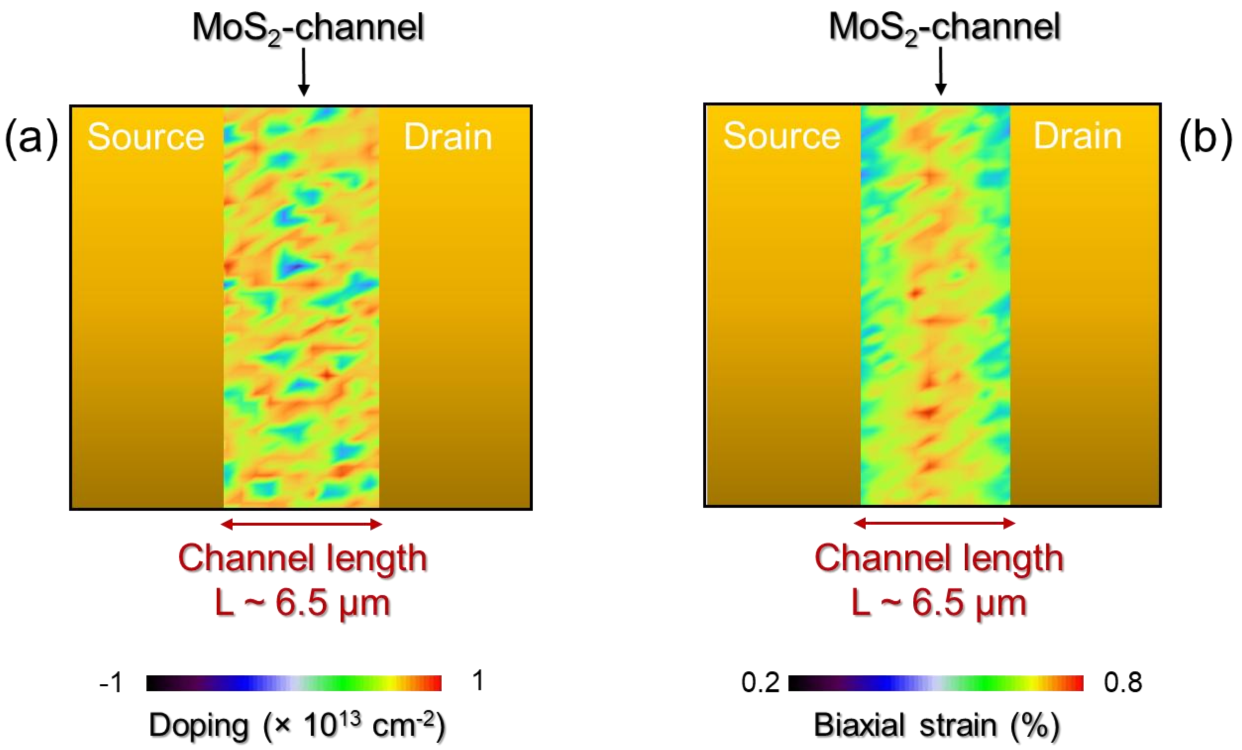

Figure S5.- Doping (a) and biaxial strain (b) maps obtained from the corresponding A_{1g} (**Fig.7**(c)) and E_{2g} (**Fig.7**(d)) maps of the main text, respectively.

References

[1] H. Kim, G. H. Han, S. J. Yun, J. Zhao, D. H. Keum, H. Y. Jeong, T. H. Ly, Y. Jin, J.-H. Park, B. H. Moon, S.-W. Kim, Y. H. Lee, Role of alkali metal promoter in enhancing lateral growth of monolayer transition metal dichalcogenides. *Nanotechnology* **2017**, *28(36)*, 36LT01.

[2] L. Seravalli, F. Esposito, M. Bosi, L. Lazzarini, F. Rossi, and F. Fabbri, L. Aversa, G.Trevisi, R. Verucchi, Built-in tensile strain dependence on the lateral size of monolayer MoS_2 synthesized by liquid precursor chemical vapor deposition, *Nanoscale* **2023**, *15*, 14669

[3] J. Jupille, A.-M. Flank, P. Lagarde, Sodium Coordination Environments in Silica Films, *Journal of the American Ceramic Society* **2002**, *85*, 1041-1046.

[4] R. A. Kalt, A. Arcifa, C. Wäckerlin, A. Stemmer, CVD of MoS_2 single layer flakes using Na_2MoO_4 – impact of oxygen and temperature–time-profile, *Nanoscale* **2023**, *15*, 18871-18882.

[5] G. J. McIntosh, A. Chan, Probing hydrogen bonding interactions and impurity intercalation in gibbsite using experimental and theoretical XANES spectroscopy, *Phys. Chem. Chem. Phys.* **2018**, *20*, 24033-24044.

[6] L. Cormier, D.R. Neuville, Development of a sequential extraction procedure for iron: Implications for iron partitioning in continentally derived particulates, *Chemical Geology* **2004**, *213*, 103–113.

[7] D. A. McKeown, G. A. Waychunas, G. E. Brown Jr, EXAFS and XANES study of the local coordination environment of sodium in a series of silica-rich glasses and selected minerals within the Na_2O, Al_2O_3, SiO_2 system, *Journal of Non-Crystalline Solids* **1985**, *74*, 325-348.

[8] X. Kong. I. Gladich, N. Fauré, E. S. Thomson, J. Chen, L. Artiglia, M. Ammann, T. Bartels-Rausch, Z. A. Kanji, J. B. C. Pettersson, Adsorbed Water Promotes Chemically Active Environments on the Surface of Sodium Chloride, *J. Phys. Chem. Lett.* **2023**, 14, 26, 6151–6156.
